# Therapeutic angiogenesis using autologous adipose-derived regenerative cells in patients with critical limb ischaemia in Japan: a clinical pilot study

**DOI:** 10.1038/s41598-020-73096-y

**Published:** 2020-09-29

**Authors:** Takeshi Katagiri, Kazuhisa Kondo, Rei Shibata, Ryo Hayashida, Satoshi Shintani, Shukuro Yamaguchi, Yuuki Shimizu, Kazumasa Unno, Ryosuke Kikuchi, Akio Kodama, Keisuke Takanari, Yuzuru Kamei, Kimihiro Komori, Toyoaki Murohara

**Affiliations:** 1 Department of Cardiology, Nagoya University Graduate School of Medicine, 65 Tsurumai-cho, Showa-ku, Nagoya, 466-8550 Japan; 2 Department of Advanced Cardiovascular Therapeutics, Nagoya University Graduate School of Medicine, 65 Tsurumai, Showa, Nagoya, 466-8550 Japan; 3 Department of Medical Technique, Nagoya University Hospital, Nagoya, Japan; 4 Department of Vascular Surgery, Nagoya University Graduate School of Medicine, Nagoya, Japan; 5 Department of Plastic and Reconstructive Surgery, Nagoya University Graduate School of Medicine, Nagoya, Japan

**Keywords:** Peripheral vascular disease, Cardiovascular diseases, Adult stem cells

## Abstract

Adipose-derived regenerative cell (ADRC) is a promising alternative source of autologous somatic stem cells for the repair of damaged tissue. This study aimed to assess the safety and feasibility of autologous ADRC implantation for therapeutic angiogenesis in patients with critical limb ischaemia (CLI). A clinical pilot study—Therapeutic Angiogenesis by Cell Transplantation using ADRCs (TACT-ADRC) study—was initiated in Japan. Adipose tissue was obtained by ordinary liposuction method. Isolated ADRCs were injected into the ischaemic limb. We performed TACT-ADRC procedure in five patients with CLI. At 6 months, no adverse events related to the TACT-ADRC were observed. No patients required major limb amputation, and ischaemic ulcers were partly or completely healed during the 6-month follow-up. In all cases, significant clinical improvements were seen in terms of rest pain and 6-min walking distance. Numbers of circulating CD34^+^ and CD133^+^ cells markers of progenitor cell persistently increased after ADRC implantation. The ratio of VEGF-A_165_b (an anti-angiogenic isoform of VEGF) to total VEGF-A in plasma significantly decreased after ADRC implantation. In vitro experiments, cultured with ADRC-conditioned media (CM) resulted in increased total VEGF-A and decreased VEGF-A_165_b in C2C12 cells, but not in macrophages. ADRC-CM also increased CD206^+^ cells expression and decreased TNF-α in macrophages. Autologous ADRC implantation was safe and effective in patients with CLI and could repair damaged tissue via its ability to promote angiogenesis and suppress tissue inflammation.

## Introduction

Critical limb ischaemia (CLI) is the most advanced stage of peripheral artery disease (PAD), Buerger disease or thromboangiitis obliterans (TAO), and collagen disease-associated vasculitis (CDV). Consequently, many patients with CLI require amputations of the affected limbs when conventional therapeutic options, including bypass surgery or endovascular treatment (EVT), fail or are not indicated^1,2^. In one-third of patients with CLI, surgical options are not suitable by the reasons that include complications, unfavourable general conditions, extensive necrosis or infection, or technical issues, such as the absence of a graftable artery^3^. Since Isner et al. reported the efficacy of gene therapy in promoting angiogenesis by using a vascular endothelial growth factor-A plasmid^4^, the concept of therapeutic angiogenesis has been considered a promising strategy for patients with CLI having no surgical options. In 2002, we reported the feasibility and safety of intramuscular autologous bone marrow-derived mononuclear cell (BM-MNC) implantation in patients with CLI in the first clinical pilot trial—Therapeutic Angiogenesis by Cell Transplantation (TACT)^5^. However, BM-MNC implantation was found to be less effective in patients with CLI caused by PAD than in those with CLI caused by TAO^6^.

Zuk et al. reported that adipose tissue contains multipotent mesenchymal stem cells (MSCs) also known as adipose-derived stem/stromal cells (ASCs) or adipose-derived regenerative cells (ADRCs) that exhibit characteristics similar to BM-MSCs^7,8^. It is believed that ADRCs represent a promising alternate source of autologous somatic stem cells for regeneration and repair of damaged tissue^9,10^. We reported that, in preclinical animal models, ADRC implantation augmented ischaemia-induced angiogenesis and maintained myocardial capillary density in response to ischaemia^11–13^. Adipose tissue can be easily and safely obtained by a common liposuction procedure in humans and is less invasive compared to BM aspiration for BM-MNC therapy. Moreover, ADRCs have been successfully used in several clinical situations^14–17^. This information prompted us to use ADRCs as an alternative cell source instead of BM-MNCs for the treatment of patients with CLI. To assess the safety and feasibility of intramuscular autologous ADRC implantation in patients with CLI, we initiated a clinical pilot study—Therapeutic Angiogenesis by Cell Transplantation using ADRCs (TACT-ADRC) study—in Japan and analysed its results (UMIN ID: UMIN000010143; 01/03/2013).

## Results

### Baseline characteristics of patients

Five patients were enrolled in this pilot study from April 2013 to November 2015. Four patients had a lower limb ischaemic lesion and one patient had an upper limb ischaemic lesion. All of them were classified as Fontaine class IV. Among all the patients, three patients had CLI caused by CDV, one patient had CLI caused by TAO, and one patient had CLI caused by a combination of PAD and CDV. Only two patients had undergone previous bypass surgery or EVT for CLI. All the patients received antiplatelet therapy. Baseline patient and lesion characteristics are shown in Table 1.

**Table 1:** Baseline patients’ and lesion characteristics.

Case	Age	Gender	Diagnosis	Affected lesion	Fontaine	ABI	SPP (mmHg)	Pain scale (NRS)	Walking distance in 6 min (m)	Comorbidities	Smoking	Previous bypass surgery or EVT
1	64	Female	SSc	Right 1^st^ toe	IV	1.06	24	3	400	None	Non-smoker	Bypass surgery
2	65	Female	SSc + PAD	Right 1^st^ and 2^nd^ toe	IV	0.55	20	3	0	Hypertension, pulmonary hypertension	Ex-smoker	EVT
3	49	Male	TAO	Left 5^th^ toe	IV	1.06	38	5	220	None	Ex-smoker	None
4	28	Female	SSc + SLE	Right dorsum of hand	IV	Unmeasured	52	8	Unmeasured	None	Non-smoker	None
5	37	Female	SLE	Left 4^th^ toe	IV	0.67	17	3	0	Renal transplantation, right limb amputation	Non-smoker	None

*ABI* ankle brachial index, *NRS* numerical rating scale, *SPP* skin perfusion pressure, *EVT* endovascular treatment, *SSc* systemic sclerosis, *PAD* peripheral artery disease, *TAO* Buerger’s disease, *SLE* systemic lupus erythematosus.

### Efficacy outcomes

**Figure 1:**
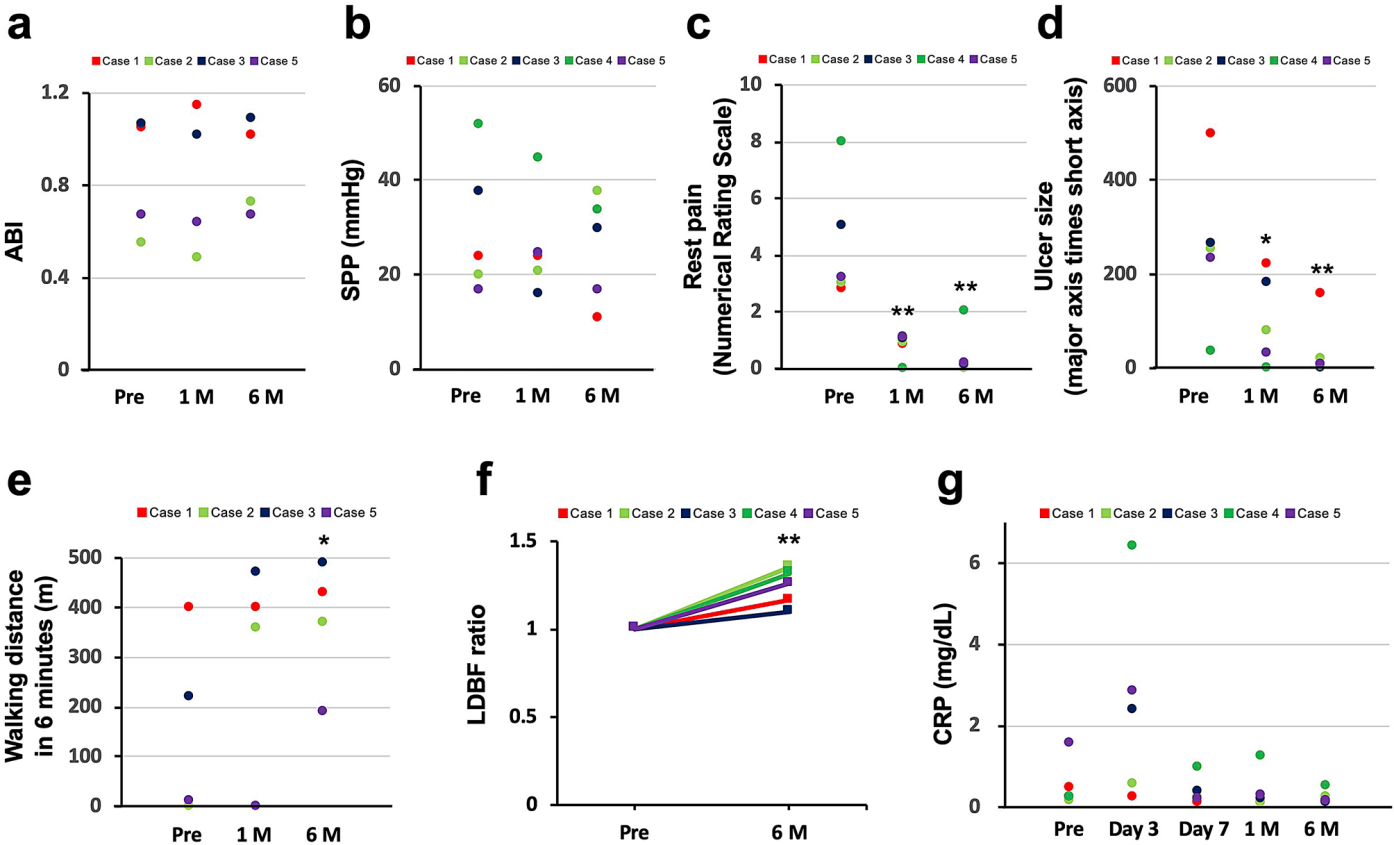
Clinical outcomes after ADRC implantation. Tissue blood perfusion indicated by ABI (**a**) and SPP (**b**) did not significantly change after ADRC implantation. Pain scale was evaluated as per the NRS (**c**) and ulcer size was calculated as the grand total of major axis length times the minor axis length (**d**) and showed significant improvement after 1 and 6 months of ADRC implantation; walking distance covered in 6 min (**e**) after ADRC implantation significantly increased 6 months after ADRC implantation. (**f**) The changes in blood perfusion levels from pre-surgery to a follow-up period of 6 months. Blood perfusion levels were assessed using a LDBF analyzer. (**g**) The levels of CRP both before and after ADRC implantation. The values shown are the means ± SEMs. * indicates *p* < 0.05 and ** indicates *p* < 0.01, compared to the pre-procedure. ADRC: adipose-derived regenerative cell, ABI: ankle brachial index, SPP: skin perfusion pressure, and NRS: numerical rating scale.

**Figure 2:**
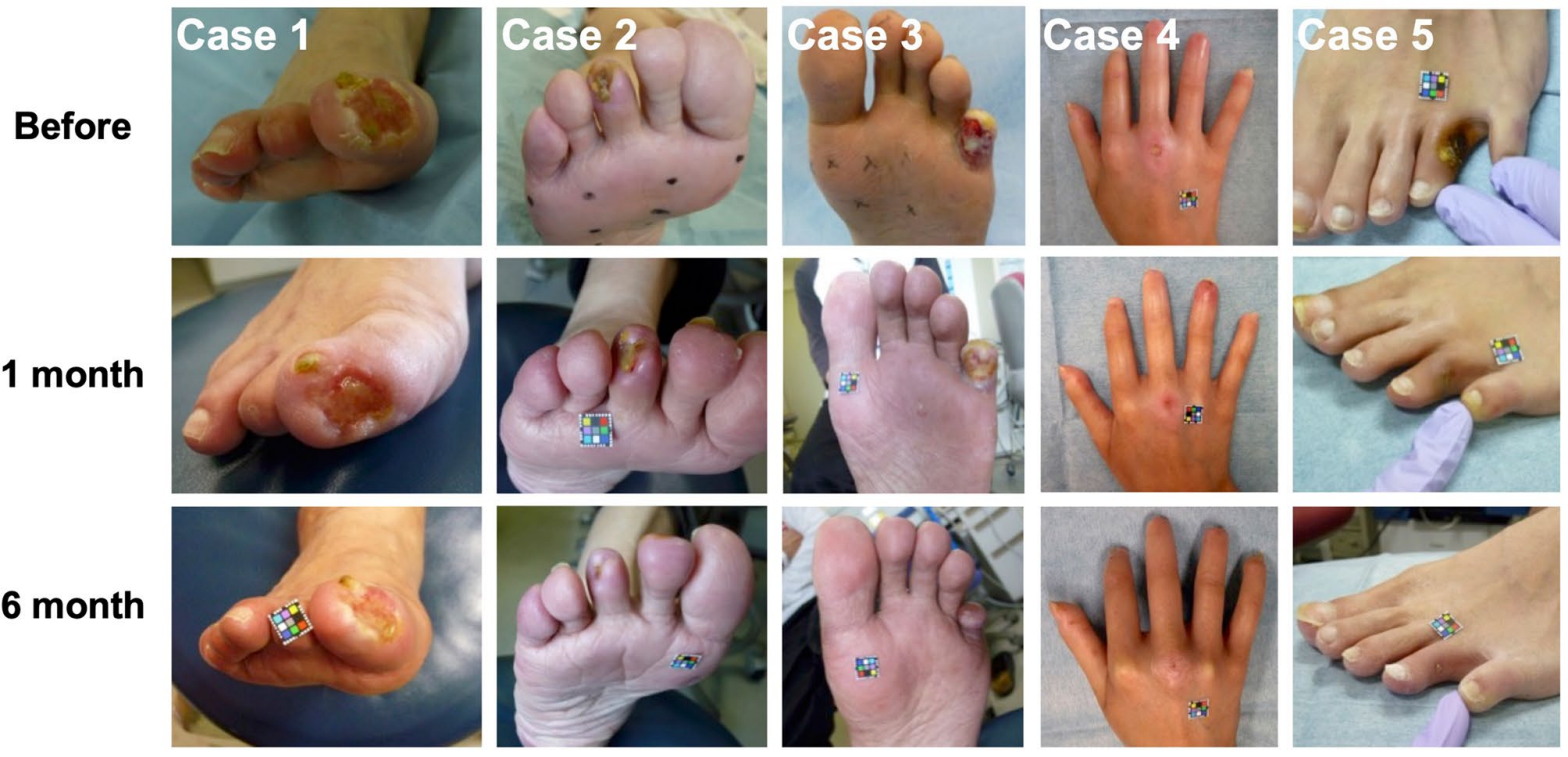
Limb salvage after ADRC implantation. Representative pictures before and after ADRC implantation (Case 1–5).

The clinical outcomes of the five patients are shown in Table 2. An average of 3.8 × 10^7^ (0.13 to 6.4 × 10^7^ cells) ADRCs were implanted intramuscularly in the ischaemic limbs. The mean cell viability was 82.3 ± 3.5%. No additional revascularization therapy was provided and no major or minor amputations were performed in any of the patients during the 6-month follow-up period, indicating that the amputation-free survival ratio (AFS) rate was 100%. Tissue blood perfusion indicated by ABI (pre: 0.84 ± 0.13,1-month: 0.83 ± 0.16, and 6-month 0.88 ± 0.10; n = 4) and SPP (pre: 14.6 ± 6.5,1-month: 11.1 ± 5.0, and 6-month 11.3. ± 5.1; n = 5) did not significantly change during the 6-month follow-up period (Fig. 1a, b). In spite of painkiller reduction or discontinuance after ADRC implantation in all the patients, the pain scale was significantly improved at 1 and 6 months after the procedure (pre: 4.4 ± 1.0,1-month: 0.8 ± 0.2; *p* < 0.05 vs. pre, and 6-month 36.0 ± 31.9; *p* < 0.01 vs. pre, n = 5) (Fig. 1c). Ischaemic ulcers and necrosis were completely healed in three patients and well improved in two patients 6 months after ADRC implantation as shown in Fig. 2. As per the results, the ulcer size diminished significantly at 1 and 6 months after the procedure (pre: 258.2 ± 164.7, 1-month: 103.8 ± 42.9; *p* < 0.05 vs. pre, and 6-month 0.4 ± 0.4; *p* < 0.01 vs. pre, n = 5) (Fig. 1d). The distance walked in 6 min also significantly increased 6 months after ADRC implantation (pre: 155.0 ± 96.7,1-month: 307.5 ± 105.0; *p* = ns vs. pre, and 6-month 370.0 ± 64.8; *p* < 0.05 vs. pre, n = 4) (Fig. 1e).

**Table 2:** Clinical outcomes at 1 and 6 months after the TACT-ADRC procedure.

Case	Implanted cell count	Major limb amputation	Minor limb amputation	Fontaine		ABI		SPP (mmHg)		Pain scale (NRS)		Walking distance in 6 min (m)		MACE
				1 M	6 M	1 M	6 M	1 M	6 M	1 M	6 M	1 M	6 M	
1	1.3 × 10^6^	None	None	IV	IV	1.15	1.02	24	11	1	0	400	430	None
2	3.7 × 10^7^	None	None	IV	IV	0.49	0.73	21	38	1	0	360	370	None
3	4.3 × 10^7^	None	None	IV	0	1.02	1.09	16	30	1	0	470	490	None
4	4.4 × 10^7^	None	None	IV	III	Unmeasured		45	34	0	2	Unmeasured		None
5	6.4 × 10^7^	None	None	IV	0	0.64	0.67	25	17	1	0	0	190	None

*ABI* ankle brachial index, *NRS* numerical rating scale, *SPP* skin perfusion pressure, *MACE* major adverse cardiovascular events.

The situation at the damage tissue was assessed using a LDBF analyzer. We measured the changes in blood perfusion levels from pre-surgery to a follow-up period of 6 months. Changes in blood flow significantly increased during the follow-up period (Fig. 1f). Furthermore, we evaluated the levels of CRP as an inflammatory marker both before and after ADRC implantation in all the five patients. CRP levels did not differ between the patients before the procedure. Serum levels of CRP persistently increased for 3 d after the TACT-ADRC procedure in all the cases, and after that, returned to levels observed before the procedure (Fig. 1g).

### Safety outcomes

All the patients undergoing therapy remained alive; overall, no severe adverse events, including MACEs (which are usually associated with liposuction and ADRC implantation), were evident among the patients (Table 2). For a few days after the procedure, all the patients experienced mild pain at the site of liposuction and cell implantation, which was easily controlled using oral painkillers, such as acetaminophens and/or NSAIDs.

### Circulating progenitor cells after ADRC implantation

**Figure 3:**
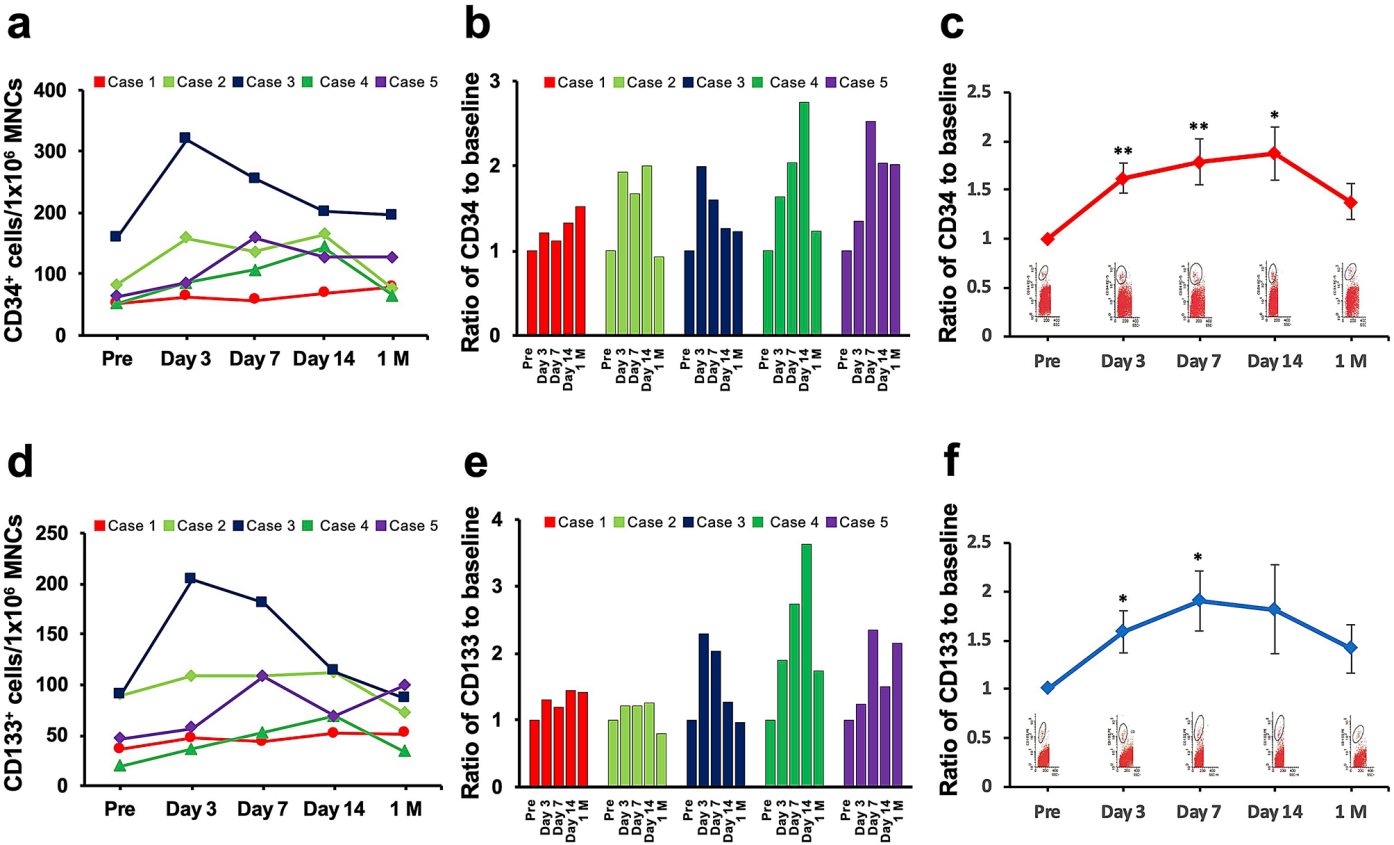
Circulating progenitor cells after ADRC implantation. FACS analysis indicating an increase in the numbers of circulating CD34^+^ (a, b and c) and CD133^+^ cells (d, e and f) in all the patients after ADRC implantation. FACS: fluorescence-activated cell sorting, ADRC: adipose-derived regenerative cell, MNCs: Mononuclear cells.

We previously reported that the numbers of circulating CD34^+^ and CD133^+^ cells increased persistently for 1 month after BM-MNC treatment in the responders^18^. We counted these numbers in peripheral blood before and after ADRC implantation by FACS analysis. FACS analysis revealed that both CD 34^+^ (Fig. 3a, b) and CD133^+^ cells (Fig. 3d, e) exhibited a tendency to increase after ADRC implantation in all the five patients. The changes in the numbers of circulating CD34^+^ cells significantly increased at days 3, 7 and 14 post treatment (Fig. 3c). Changes in CD133^+^ cells also significantly increased at days 3 and 7 (Fig. 3f).

### Circulating VEGF isoforms levels after ADRC implantation

**Figure 4:**
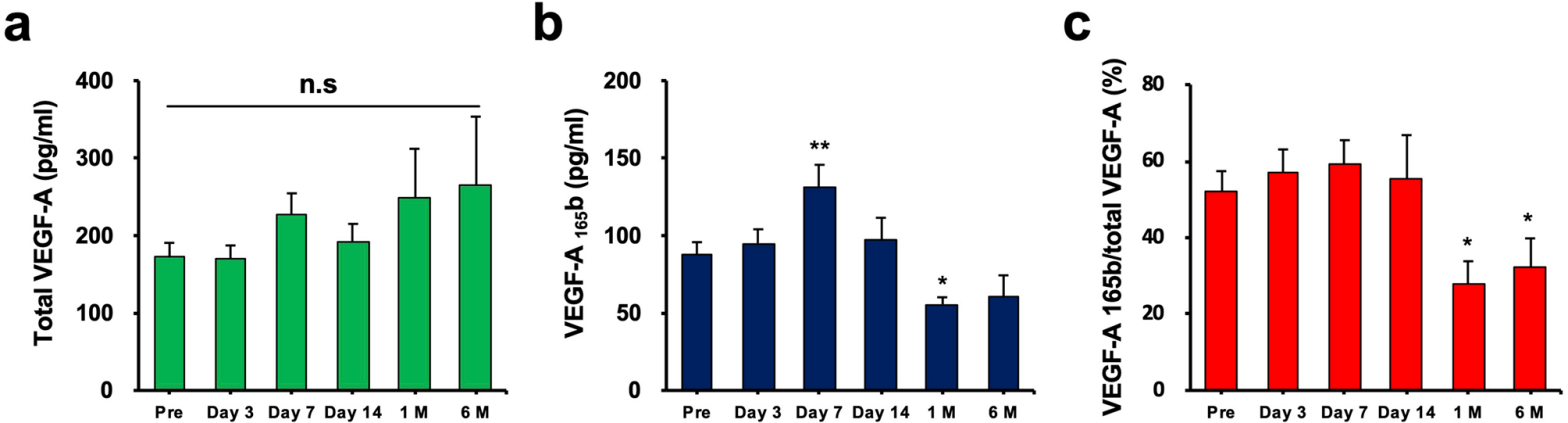
Serum VEGF isoforms levels after ADRC implantation. Serum levels of total VEGF-A did not alter significantly (**a**), but levels of VEGF-A_165_b decreased significantly at 1 month after ADRC implantation (**b**); the ratio of VEGF-A_165_b to total VEGF-A significantly decreased at 1 and 6 months after ADRC implantation (**c**). The values shown are the means ± SEMs. * indicates *p* < 0.05 and ** indicates *p* < 0.01, compared to the pre-procedure. VEGF: vascular endothelial growth factor.

Recently, we reported that impaired vascularization in patients with PAD is associated with elevated levels of VEGF-A_165_b, an antiangiogenic VEGF-A alternative splice variant, and reduced levels of the proangiogenic VEGF-A_165_a splice isoform^19^. Therefore, we evaluated the levels of the circulating VEGF-A isoforms, including total VEGF-A and VEGF-A_165_b, before and after the ADRC implantation using ELISA. Total VEGF-A and VEGF-A_165_b transiently increased on day 7 after the TACT-ADRC procedure; this was considered to be the direct effect of the procedure (Fig. 4a, b). Although serum levels of total VEGF-A did not significantly alter (Fig. 4a) during the 6-month follow-up period, levels of VEGF-A_165_b significantly decreased by 36.2% after 1 month of ADRC implantation (*p* < 0.05 vs. pre, n = 5) (Fig. 4b); the ratio of VEGF-A_165_b to total VEGF-A significantly decreased by 46.5% at 1 month (*p* < 0.05 vs. pre) and by 38.3% at 6 months after ADRC implantation (*p* < 0.05 vs. pre, n = 5) (Fig. 4c).

### Effect of ADRC on the expression of VEGF isoforms in macrophages and C2C12 cells

**Figure 5:**
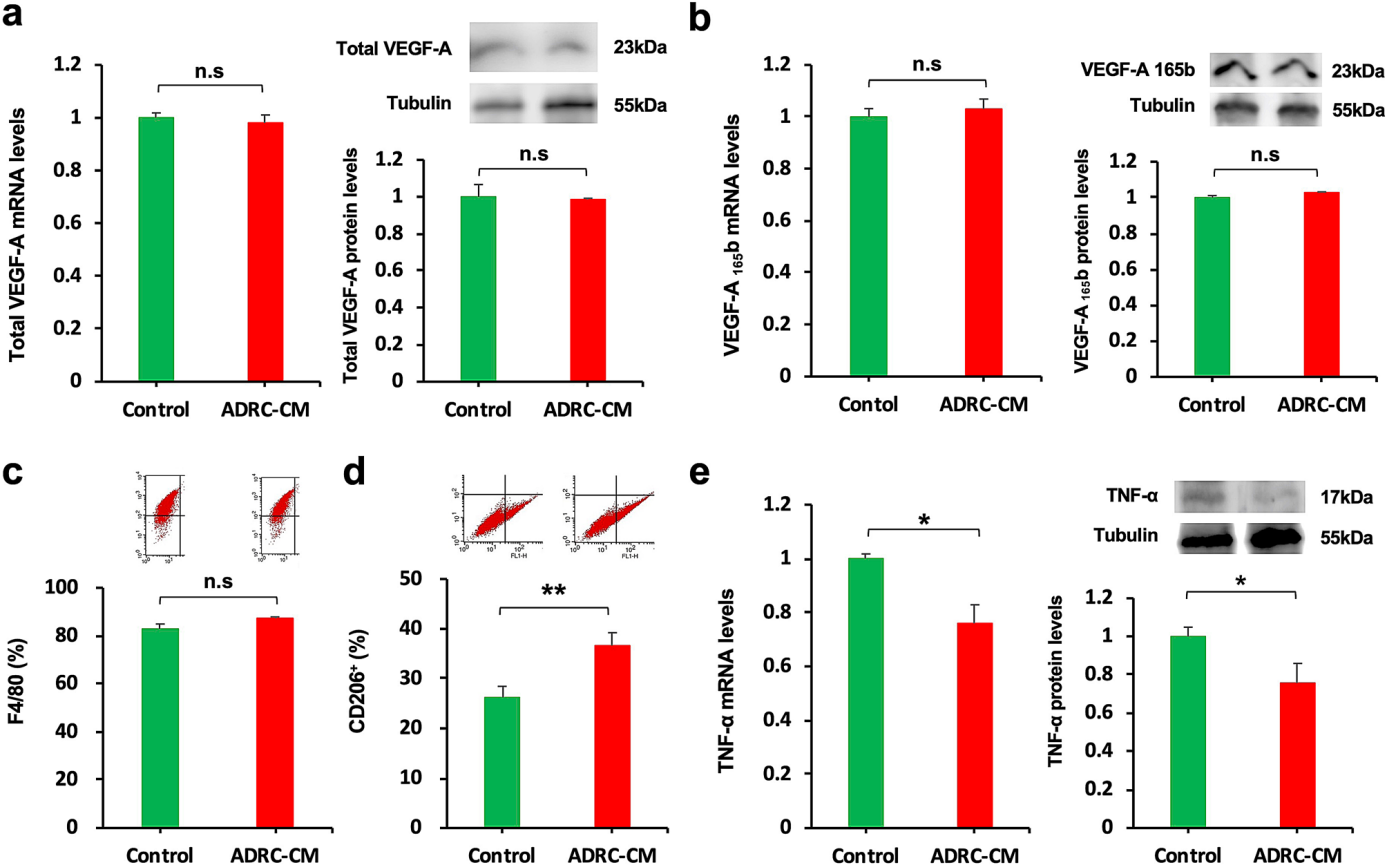
Effects of ADRC on the expression of VEGF isoforms in macrophages. Effect of ADRC-CM on the expression of total VEGF-A (**a**), VEGF-A_165_b (**b**), F4/80 (**c**), CD206^+^ cells (**d**), and TNF-α (**e**) in peritoneal macrophages from WT mice. Macrophages were pretreated with ADRC-CM or control medium for 24 h. mRNA expression of total VEGF-A, VEGF-A_165_b, and TNF-a was measured by real-time PCR and expressed relative to Gapdh levels (n = 12 in each group). The levels of proteins VEGF-A, VEGF-A_165_b, and TNF-a in the ADRC-CM treated cells were assessed by western blotting analysis (n = 4 in each group). The expression of F4/80 and CD206^+^ cells was analyzed by flow cytometric analysis (n = 4 in each group).

To investigate the effect of ADRC on VEGF isoform alterations at the cellular level, an in vitro study was performed using peritoneal macrophages from WT mice. Treatment of macrophages with ADRC-CM for 24 h did not affect the expression of total VEGF-A, VEGF-A_165_b, and F4/80 (Fig. 5a–c). In contrast, the ADRC-CM treatment increased the expression levels of CD206^+^ cells, an anti-inflammatory M2 macrophage marker, compared to the control medium (Fig. 5d). The expression of the inflammatory M1 macrophage marker, TNF-α, were downregulated in the macrophages cultured with the ADRC-CM (Fig. 5e).

**Figure 6:**
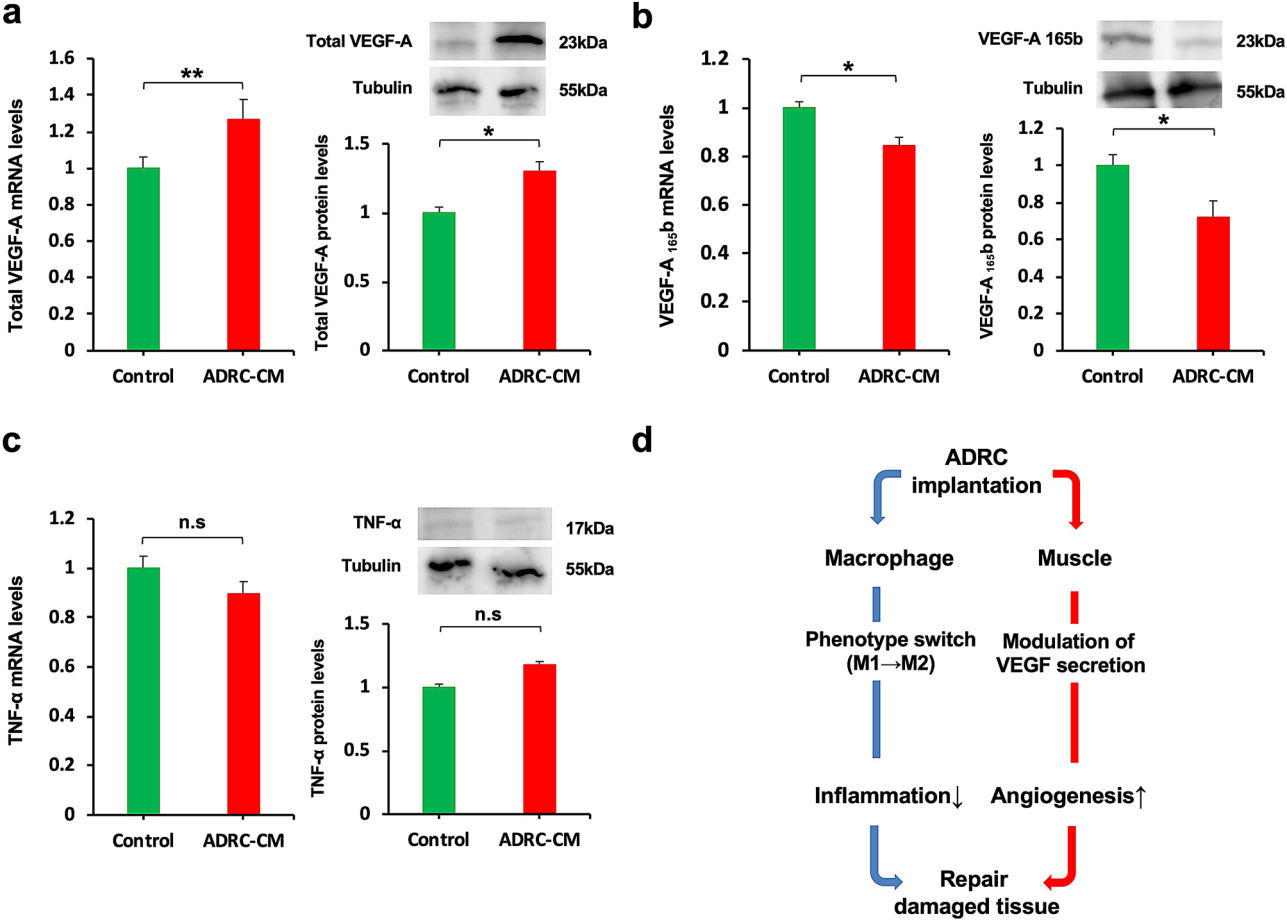
Effects of ADRC on the expression of VEGF isoforms in C2C12 cells. Effect of ADRC-CM on the expression of total VEGF-A (**a**), VEGF-A_165_b (**b**), and TNF-α (**c**) in differentiated C2C12 mouse skeletal muscle cells. C2C12 cells were pretreated with ADRC-CM or control medium for 24 h. The mRNA expression of molecules was measured by real-time PCR and expressed relative to *Gapdh* levels (n = 8 in each group). The levels of proteins VEGF-A, VEGF-A_165_b, and TNF-a in the ADRC-CM treated cells were assessed by western blotting analysis (n = 4 in each group). (**d**) Proposed scheme for the effects of ADRC therapy in the repair of damaged tissue. Results are presented as the mean ± SEM. * indicates *p* < 0.05 and ** indicates *p* < 0.01.

We next assessed the expression of VEGF isoforms in differentiated C2C12 mouse skeletal muscle cells. Treatment of C2C12 myotubes with ADRC-CM for 24 h significantly increased the expression of total VEGF-A (Fig. 6a). In contrast, the expression of anti-angiogenic VEGF-A_165_b decreased in C2C12 cells cultured with ADRC-CM compared to the control medium (Fig. 6b). No significant differences were observed between the TNF-α mRNA levels in the C2C12 cells cultured with ADRC-CM and in those cultured in the control medium (Fig. 6c). Thus, ADRC is likely to act on macrophages to promote switching to an anti-inflammatory phenotype and on skeletal muscle to regulate the secretion of VEGF isoforms (Fig. 6d).

## Discussion

To our knowledge, the current study is the first report of fresh isolated ADRC therapy with the Celution system in patients with CLI who have no available surgical revascularization options. We previously reported that ADRC implantation augmented ischaemia-induced angiogenesis in hindlimb ischaemia model in rodents^11,12^; subsequently, we performed a safety confirmation examination by liposuction, ADRC isolation using the Celution system, and ADRC implantation in pig (unpublished data). Finally, we developed a therapeutic angiogenesis protocol, termed the TACT-ADRC procedure, involving the implantation of autologous fresh ADRCs isolated using the Celution system in patients with CLI in Japan. It has been previously reported that ADRCs isolated with the Celution system consist of heterogeneous cell populations, which include MSCs, mature and progenitor endothelial cells (ECs), vascular smooth muscle cells, CD45^+^ haematopoietic cells, resident tissue monocyte/macrophages, pericytes, and preadipocytes^20^. Since ASCs or ADRCs were first found in the adipose tissue, they have been known to be a promising alternative source of autologous somatic stem cells for the regeneration and repair of damaged tissue^21^. In numerous animal models, ADRCs exhibited angiogenic effects via the ability of EC lineage differentiation^22,23^ and/or secretion of angiogenic cytokine and chemokines, such as VEGF-A, HGF, bFGF, and SDF-1^9,11,12,24^. Furthermore, ADRCs have already been successfully used in the treatment of several diseases in clinical fields, including Crohn’s disease-associated fistulas^17^, breast reconstruction after partial mastectomy^16^, osteogenesis for bone defects^15,25^, stress urinary incontinence^14,26^, and ischaemic heart disease^27,28^. Furthermore, the safety and feasibility of ADRCs isolated using the Celution system have already been confirmed in several clinical studies^14,26–28^.

The goal of therapy for patients with CLI is the prevention of limb amputation and improvement of symptoms, which eventually improve the quality of life and survival. Revascularization therapy, including bypass surgery and EVT, is the primary approach used to improve CLI symptoms; however, approximately one-third of patients with CLI cannot undergo surgical revascularization^3^. To treat such patients, implantation therapy using somatic stem cells, including BM-MNCs, was developed almost two decades ago; the findings from the initial clinical studies that investigated BM-MNC implantation, including the TACT study, indicated that intramuscular autologous BM-MNC therapy was a potentially effective and safe therapeutic strategy for patients with CLI who had no available surgical options^29,30^. However, the findings of the long-term outcome evaluations in the TACT study revealed that BM-MNC implantation was less effective in patients with CLI caused by PAD than in patients with CLI caused by TAO and CDV^6,31^. Also, bone marrow harvest is invasive, therefore alternative stem/progenitor cell sources for therapeutic angiogenesis have been explored by various research groups, including ours. In the current study, non-cultured ADRC implantation demonstrated 100% AFS rate and significant improvements in rest pain, ulcer size, and walking distance in five patients with CLI caused by TAO or CDV but showed no changes in ABI and SPO_2_. These results appear to be consistent with previous reports of the long-term outcomes of the TACT study. No severe adverse events were observed; no patients died or required major and minor limb amputation during 6-month follow-up period after ADRC implantation. As far as we know, there are only two other clinical studies of ASC therapy for CLI previously reported by a Korean group^32^ and a French group^33^, which describe the safety and efficacy of culture-expanded ASC implantation in patients with CLI. Lee et al. demonstrated that multiple intramuscularly culture-expanded ASC (3 × 10^8^ cells) injections significantly improved ulcer healing, pain rating scale, and claudication walking distance in 15 patients^32^; Bura et al. also injected culture-expanded ASCs (1 × 10^8^ cells) intramuscularly into the gastrocnemius and anterior compartment of the ischaemic leg in seven patients, resulting in CLI grade improvement and increased TcPO2^33^. Although it is difficult to directly compare our results with those of the others because the severity and cause of CLI are different among the studies, our results are comparable to those of the other studies. Furthermore, we believe that our prodcedure has several advantages over the above mentioned studies. Firstly, we used fresh ADRCs automatically and aseptically isolated using the commercially available closed centrifugal cell separator system in the TACT-ADRC procedure; it takes only approximately 4 h from general anaesthesia to the end of the procedure. Meanwhile, a few weeks of culture are necessary to obtain a sufficient number of ADRCs for the treatment used by the other groups. Secondly, the non-culture method offers greater advantages with regard to concerns about the contamination of pathogens, chemical regents, and biomaterials, such as FBS and trypsin. Finally, the cost of treatment using our procedure is much lower than a procedure with culture-expanded ASCs, because a specific cell processing room according to GCP standards is unnecessary. Very recently, the results of the randomized, double-blind, parallel-group, placebo-controlled phase 3 ADMIRE-CD study for the treatment of patients with complex perianal fistula in Crohn’s disease with ASCs have been reported^17^. This trial revealed that a single injection of allogeneic expanded ASCs (Cx601) (1.2 × 10^8^ cells) into the tissue adjacent to fistula tracts and internal openings achieved a combined remission of 50% in the Cx601 group compared to 34% in the placebo group; moreover, treatment-related adverse events occurred more frequently in the placebo group compared to the Cx601 group. If allogenically expanded ADRCs are to be used safely and effectively in patients with CLI, it would need a minimally invasive and quick strategy; however, studies are needed to confirm this in future.

EPC mobilization and functions are reduced in patients with arteriosclerosis risk factors^34,35^, chronic ischaemic cardiomyopathy^36^, and diabetes^37^ and in those undergoing haemodialysis. We reported that ADRC implantation augmented ischaemia-induced angiogenesis via an increase in EPC mobilization by the paracrine effect of VEGF and SDF-1^11^; the number of circulating progenitor cells, such as CD34^+^ and CD133^+^ cells, persistently increased in responders up to 1 month after BM-MNC implantation^18^. However, there is no report about circulating progenitor kinetics after ADRC implantation in humans. In the current study, we demonstrate for the first time, to our knowledge, that circulating progenitor cells show an increase after ADRC implantation, which is consistent with our previous reports. These results may be a part of the mechanism of the angiogenic and cytoprotective effects of ADRC implantation.

Possible mechanisms of regeneration and repair of damaged tissue by ADRCs are mainly considered to depend on the paracrine effect of cytoprotective and angiogenic factors, including proteins of VEGF family^11,38^, HGF^9^, bFGF^13^, and SDF-1^11,39^, rather than transdifferentiation of these cells into the cells, which result in angiogenic, anti-inflammatory and anti-immunomodulatory, and anti-apoptotic action^12,17,40^. VEGF-A is a strong angiogenic cytokine^41^ that can mobilize EPCs from BM; however, plasma levels of VEGF-A are known to increase in patients with PAD^42^. Although this phenomenon was called “the angiogenesis paradox”, we have recently clarified that impaired vascularization in patients with PAD is associated with elevated levels of antiangiogenic VEGF-A_165_b, a VEGF-A splice isoform, and reduced levels of the proangiogenic VEGF-A_165_a splice isoform^19^. In the current study, we found that levels of VEGF-A_165_b significantly decreased after ADRC implantation, but total VEGF-A levels did not change. In the in vitro experiments, ADRC-associated VEGF was secreted in skeletal muscle cells but not in macrophages. In contrast, macrophages switched to an anti-inflammatory phenotype upon exposure to ADRCs. Collectively, ADRCs could repair damaged tissue by the promotion of angiogenesis via modulation of VEGF secretion in skeletal muscle cells and suppression of inflammatory reactions in macrophages (Fig. 6d).

In our study, we did not examine the location of the implanted ADRCs during chronic phase, because it would be difficult and unethical to immunohistochemically investigate ADRC-implanted limbs of patients. Previously, we examined how long the transplanted cells survived in the ischemic tissue in vivo experiments^11^. We confirmed that implanted GFP-mice derived ADRCs could survive in ischemic tissues for at least 28 postoperative days. The transplanted cells were located in nearby vessels. In addition, we found that ADRCs differentiated into pericytes but not into endothelial cells. Thus, we think that at least some part of the transplanted ADRCs differentiated into pericytes.

This study has several limitations. The TACT-ADRC study was a single arm, non-randomized, open-labelled, historical control clinical pilot study performed in a single institute. This study consisted of a very small number of patients and did not include control groups, which is an obvious weakness of the current study. Moreover, the basal diseases were different among the patients. Therefore, it is difficult to ascertain if the TACT-ADRC procedure is safe and effective only from the results from this pilot study. Especially, we should elucidate whether this therapy promotes unfavorable angiogenesis associated with tumors under long-term follow-up. In addition, fresh ADRCs were isolated from the patients themselves. The effect of those factors such as age and health condition (medical history) on the disease outcome is unknown. In our preliminary data, certain cytokine secretions varied by age, gender, and metabolic parameters as determined by using a cytokine Array kit. Thus, future studies will be required to clarify the effect of these factors, such as age and health condition, on the disease outcome.

On the other hand, our trial, as a clinical pilot study, did not use placebo control patients from a humane standpoint, and then this study protocol was approved by the Ministry of Health, Labour and Welfare in Japan under these terms. All patients who received this ADRC implantation treatment had severe CLI, and had no available alternative surgical options. They had planned to undergo amputation if this treatment was not done. In fact, the two patients who were not eligible for this treatment had a poor prognosis within 6 months (data not shown). Thus, our results suggest that the performance of ADRC implantation might be safe and effective for patients with critical limb ischemia, although this study was preliminary and did not have a placebo control group.

In Japan, a new regulation, “The Act on the Safety of Regenerative Medicine”, regarding regenerative medicine came into force on November 25, 2014. Upon the enforcement of the new law, to increase the number of enrolled patients, we slightly modified the protocol of the TACT-ADRC procedure and expanded this study into a multicentre study, thus including eight institutes, on December 2015, in Japan. However, this multicentre study is still a single arm, non-randomized, open-labelled study. Moreover, a larger number of patients is necessary to validate the effect of the TACT-ADRC procedure and analyse the requirements of the responders. We were unable, however, to enrol more patients into the original TACT-ADRC study in a single institute because of the reasons described above. Ultimately, a well-designed, multicentre, double-blinded RCT that involves a large number of patients is needed to validate the effects of ADRC implantation in future.

## Conclusions

We developed a therapeutic angiogenesis procedure involving the implantation of freshly isolated autologous ADRCs in patients with CLI. We also confirmed that autologous ADRC implantation is safe and effective for achievement of clinical therapeutic angiogenesis in five patients with CLI and that ADRC is likely to repair damaged tissue via its ability to promote angiogenesis and suppress inflammation. Hence, the TACT-ADRC procedure could be used as an alternative and feasible strategy for patients with CLI.

## Methods

### Study design

The TACT-ADRC study is an investigator-initiated, single centre, single arm, non-randomized, open-labelled clinical study conducted in Nagoya University Hospital, Japan. The target disease is CLI, which was defined as Fontaine stage III and IV due to PAD, TAO, or CDV. All procedures in studies involving human participants were performed in accordance with the ethical standards of the institutional or national research committee and with the 1964 Declaration of Helsinki and its later amendments or comparable ethical standards. The study protocol was approved by the Ministry of Health, Labor and Welfare in Japan and the ethics committee in Nagoya University Graduate School of Medicine, Japan in 2012. This study was registered on UMIN Clinical Trials Registry (UMIN ID: UMIN000010143; 01/03/2013).

### Patients

**Figure 7:**
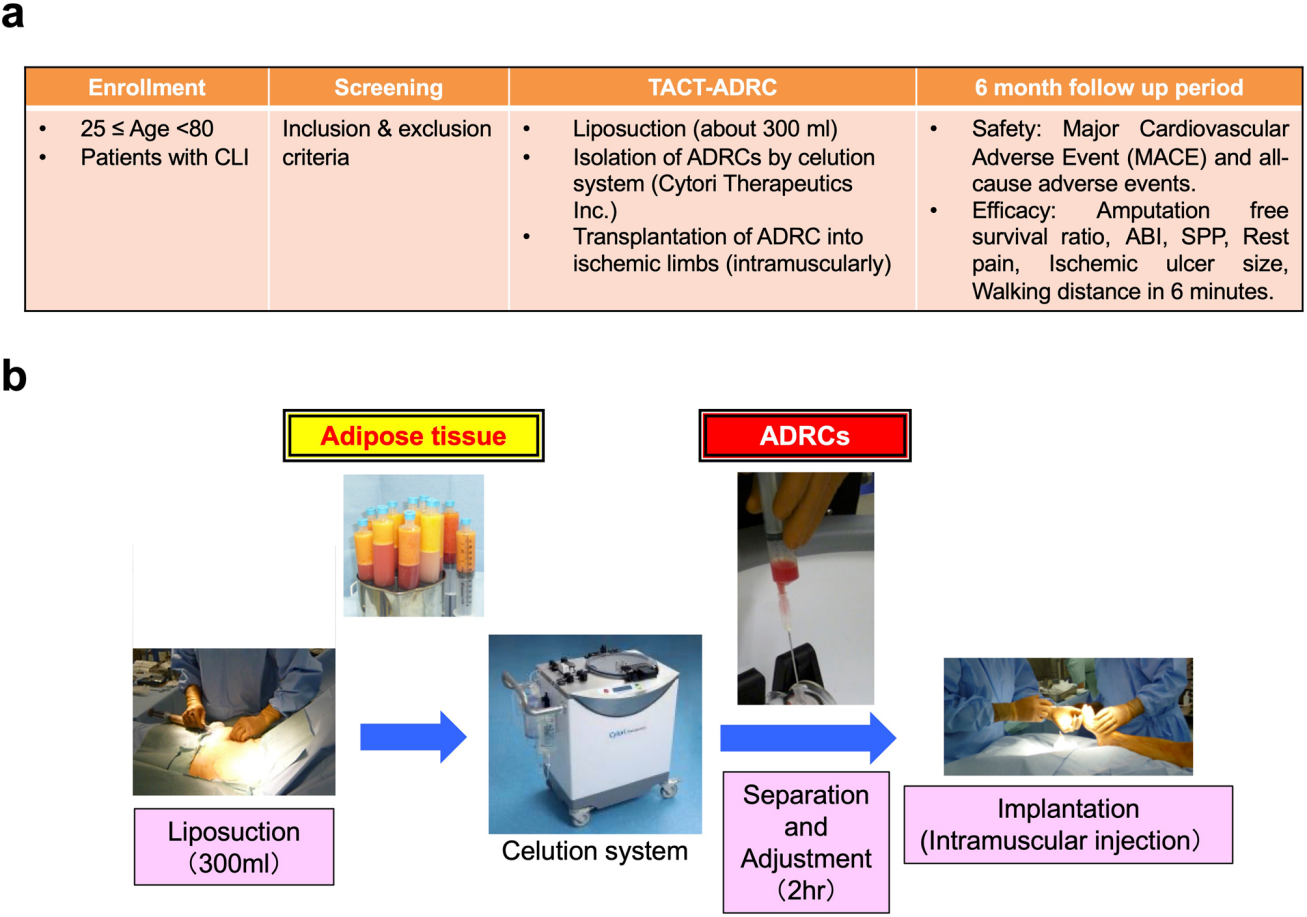
Therapeutic angiogenesis using ADRCs in patients with CLI. (**a**) Outline of protocol for the TACT-ADRC study. TACT: therapeutic angiogenesis by cell transplantation, ADRC: adipose-derived regenerative cell, CLI: critical limb ischaemia, ABI: ankle brachial index, SPP: skin perfusion pressure, MACE: major adverse cardiovascular event. (**b**) Flowchart of the TACT-ADRC procedure.

Patients with CLI (defined as Fontaine stages III and IV), who could not undergo surgical revascularization, qualified for ADRC implantation. All patients underwent pre-checks to determine whether they fulfilled any of the exclusion criteria: age > 80 years; insufficient amount of adipose tissue; life expectancy < 1 year; untreated coronary artery diseases or cerebrovascular diseases; clinical or laboratory signs of chronic or acute inflammation; a past (5 years) or current history of neoplasms, diabetes with untreated retinopathy, severe liver or kidney dysfunction, including haemodialysis, severe anaemia or haematopoietic disease; pregnancy or possibility of pregnancy; or the refusal or inability to provide informed consent (Fig. 7a). We obtained written informed consent from all the patients.

### Procedures

While the patients were under general anaesthesia, about 300 ml of adipose tissue was obtained by liposuction from the subcutaneous abdominal and/or femoral sections by well-trained plastic surgeons. Concentrated ADRCs (5 ml) were automatically and aseptically isolated using a commercially available closed centrifugal cell separator system (Celution system, Cytori Therapeutics, Inc., Austin, TX, USA)^14,20^ with adipose digesting enzyme product (Celase, Cytori Therapeutics, Inc.) in approximately 2 h; subsequently, total cell number and viability were counted using an automated cell counter (NucleoCounter NC-100, M&S TechnoSystems, Inc., Osaka, Japan) (Fig. 7b). Thus, the weight of adipose tissue, but not the number of cells, is standardized in all the five patient cases in this study. Isolated ADRCs were diluted with lactated Ringer’s solution to a total volume of 50 ml; the diluted ADRC solution was injected into the gastrocnemius or hand of the ischaemic limb, 0.5–1.0 ml in a 3 × 3 cm grid using 26-gauge needle according to the method used for BM-MNCs implantation (50–70 sites, 1–1.5 cm deep)^5^. Antiplatelet agent intake was stopped 3–5 days before liposuction depending on the type of agent and restarted 3–5 days after cell implantation depending on the situation of haemostasis.

### Endpoints

The primary outcome was the amputation-free survival ratio (AFS) during the 6-month follow-up period after the TACT-ADRC procedure. The secondary endpoints were evaluated by ABI (ankle biracial index) and skin perfusion pressure (SPP); the changes in rest pain were evaluated using the numerical rating scale (NRS). Moreover, ulcer size (grand total of the minor axis times longer axis) and walking distance in 6 min in patients with lower limb lesions were assessed. Pre-procedural and follow-up examinations at 1 and 6 months after TACT-ADRC procedure were performed. The safety of the therapy was assessed in the context of major adverse cardiovascular events (MACEs) and all-cause adverse events during the 6-month follow-up period after the TACT-ADRC procedure (Fig. 6a). The MACEs were defined as death, non-fatal myocardial infarctions, decompensated heart failure, and stroke.

### Laser Doppler blood flow analysis

The situation at the damage tissue was assessed using a laser Doppler blood flow (LDBF) analyzer (Moor LDI; Moor Instruments, Devon, United Kingdom). Immediately before surgery and on postoperative 6 months, LDBF analysis were performed on the gastrocnemius or hand of the ischemic limb. Blood flow was displayed as changes in the laser frequency using different color pixels. After scanning, stored images were analyzed to quantify blood flow^5,43^.

### Circulating progenitor cell assay

The number of circulating progenitor cells expressing CD34 or CD133 was counted by fluorescence-activated cell sorting (FACS) analysis pre-procedure and at 3 days and 1-, 2-, 4-, and 24-weeks after the ADRC treatment, as previously described^18^.

### Circulating VEGF isoforms assay

To measure the circulating levels of total VEGF-A and VEGF-A_165_b, an anti-angiogenic cytokine, blood samples were obtained from the five patients pre-procedure and at 3 days and 1-, 2-, 4-, and 24-weeks after ADRC treatment. Total circulating serum levels of total VEGF-A and VEGF-A_165_b were measured using an ELISA kit (Human VEGF Quantikine ELISA Kit, DVE00, R&D, Human Vascular Endothelial Growth Factor-_165_b ELISA Kit, MBS720132, MyBiosource, San Diego, CA, USA) according to the manufacturer's instructions and as previously described^44^.

### Cell culture

All the protocols were approved by the Institutional Animal Care and Use Committee of Nagoya University School of Medicine. Our study conformed to the Regulation and Guidelines of Animal Care and Use in Japan and Nagoya University (https://www.care.nagoya-u.ac.jp/statute.html). Freshly isolated ADRCs from the inguinal fat pads of C57BL/6 J mice (8 to 10 weeks old, Charles River Laboratories, Wilmington, MA, USA) were cultured in DMEM containing 10% foetal bovine serum (FBS) until they reached previously described confluence^11^. The medium was then switched to DMEM with 2% FBS. ADRC-conditioned media (CM) was collected 24 h after medium change. Macrophages (1 × 10^6^ cells/well) were collected from the peritoneal cavities of thioglycolate-injected C57BL/6J mice. C2C12 mouse myoblasts (American Type Culture Collection, Virginia, USA) were cultured as described elsewhere. Macrophages and C2C12 cells were cultured in ADRC-CM for 24 h as previously described^12,45^. Total RNA was isolated from cultured macrophages and C2C12 cells, and quantitative real-time RT-PCR analysis of total VEGF-A, VEGF-A_165_b, TNF-α, and Gapdh transcripts was performed as previously described^19,46^. Primers used were 5′-CAGAAAATCACTGTGAGCCTTGTT-3′ and 5′-ATTAAGGACTGTTCTGTCAA-3′ for mouse total VEGF-A, 5′-CAGAAAATCACTGTGAGCCTTGTT-3′ and 5′-GGTGAGAGGTCTGCAAGTACGTT-3′ for mouse VEGF-A_165_b, 5′-AGCCCCCAGTCTGTATCCTT-3′ and 5′-CTCCCTTTGCAGAACTCAGG-3′ for mouse TNF-α, and 5′-ATGGTGAAGGTCGGTGTG-3′ and 5′-ACCAGTGGATGCAGGGAT-3′ for mouse Gapdh. In some experiments, macrophages were incubated for 30 min with the following anti-mouse antibodies: F4/80-PE (clone BM8, BioLegend), CD206- FITC (clone C068C2, BioLegend), and analyzed by flow cytometric analysis. For western blot analysis, cell samples were prepared in lysis buffer containing 1 mM PMSF (Sigma). The protein concentration was calculated using a BCA protein assay kit (Thermo Scientific). The equal amounts of proteins were separated by denaturing SDS-PAGE. Proteins were transferred onto PVDF membrane (Bio Rad) and probed with the primary antibody followed by incubation with the HRP-conjugated secondary antibody. ECL plus system (GE Healthcare) were used for detection of the protein signa^38^. The expression level was determined by measurement of the corresponding band intensities by using Image J software, and the relative values were expressed relative to α-tubulin signal. Antibodies to VEGF-A, VEGF-A_165_b, TNF-a and α-tubulin were purchased from Cell Signaling Technology (MA, USA). VEGF-A antibody was purchased from Upstate Biotechnology.

### Statistical analyses

All data are expressed as the mean ± SEM. Statistical significance was evaluated using unpaired Student’s *t*-test for comparisons between two means and repeated measures analysis of variance (ANOVA) for comparisons among three or more means^38^. In case of a significant observation in ANOVA, Dunnett’s test was performed as a post-hoc analysis. A value of *p* < 0.05 was considered statistically significant.

## Supplementary information

Supplementary file 1.

Supplementary file 2.
