# Supplementary material for: Therapeutic angiogenesis using autologous adipose-derived regenerative cells in patients with critical limb ischaemia in Japan: a clinical pilot study
Source: Sci Rep. 2020 Sep 29;10:16045. doi: 10.1038/s41598-020-73096-y (PMC7525513; doi:10.1038/s41598-020-73096-y)
Supplement: Supplementary file 1 [file 41598_2020_73096_MOESM1_ESM.pdf]

Research protocol

Therapeutic Angiogenesis using Autologous Adipose-derived Regenerative Cells in Patients with Critical Limb Ischaemia in Japan: A Clinical Pilot Study

Takeshi Katagiri[1], Kazuhisa Kondo[1], Rei Shibata[2], Ryo Hayashida[1], Satoshi Shintani[1], Shukuro Yamaguchi[1], Yuuki Shimizu[1], Kazumasa Unno[1], Ryosuke Kikuchi[3], Akio Kodama[4], Keisuke Takanari[5], Yuzuru Kamei[5], Kimihiro Komori[4] and Toyoaki Murohara[1]

[1] Department of Cardiology, Nagoya University Graduate School of Medicine, Nagoya, Japan

[2] Department of Advanced Cardiovascular Therapeutics, Nagoya University Graduate School of Medicine, Nagoya, Japan

[3] Department of Medical Technique, Nagoya University Hospital, Japan.

[4]Department of Vascular Surgery, Nagoya University Graduate School of Medicine, Nagoya, Japan

[5] Department of Plastic and Reconstructive Surgery, Nagoya University Graduate School of Medicine, Nagoya, Japan

Project summary

Adipose-derived regenerative cell (ADRC) is a promising alternative source of autologous somatic stem cells for the repair of damaged tissue. This study aim to assess the safety and feasibility of autologous ADRC implantation for therapeutic angiogenesis in patients with critical limb ischaemia (CLI). A clinical pilot study—Therapeutic Angiogenesis by Cell Transplantation using ADRCs (TACT-ADRC) study—is initiated in Japan. Patients with CLI (defined as Fontaine stages III and IV), who could not undergo surgical revascularization, qualify for ADRC implantation. ADRCs are automatically and aseptically isolated using a commercially available closed centrifugal cell separator system (Cytori Therapeutics, Inc.) Isolated ADRCs are injected into the ischaemic limb. We assess the safety (major cardiovascular adverse event and all-cause adverse events) and feasibility (amputation free survival ratio, ABI, SPP, rest pain, ischemic ulcer size, walking distance in 6 minutes). This study is registered on UMIN Clinical Trials Registry (UMIN ID: UMIN000010143; 01/03/2013).

General information

1) Protocol title: Therapeutic Angiogenesis by Cell Transplantation using ADRCs (TACT-ADRC)

2) Protocol identifying number: UMIN000010143

3) Date: 01/03/2013

4) Name and address of the sponsor/funder: This study was supported, in part, by the Japanese Circulation Society Translational Research Grant 2010-2013 and Japan Agency for Medical Research and Development (AMED) (2014-2015).

5) Name and title of the investigator:

Toyoaki Murohara, MD, PhD (Professor).

Department of Cardiology, Nagoya University Graduate School of Medicine, 65 Tsurumai-cho, Showa-ku, Nagoya 466-8550, Japan.

Tel: +81-52-744-2149 Fax: +81-52-744-2210

Email address: murohara@med.nagoya-u.ac.jp

6) Name and address of the institutions involved in the research:
Nagoya University Graduate School of Medicine, 65 Tsurumai-cho, Showa-ku, Nagoya 466-8550, Japan.

Rationale & background information

Zuk et al. reported that adipose tissue contains multipotent mesenchymal stem cells (MSCs) also known as adipose-derived stem/stromal cells (ASCs) or adipose-derived regenerative cells (ADRCs) that exhibit characteristics similar to BM-MSCs[1,2]. It is believed that ADRCs represent a promising alternate source of autologous somatic stem cells for regeneration and repair of damaged tissue[3,4]. We reported that, in preclinical animal models, ADRC implantation augmented ischaemia-induced angiogenesis and maintained myocardial capillary density in response to ischaemia[5-7]. Adipose tissue can be easily and safely obtained by a common liposuction procedure in humans and is less invasive compared to BM aspiration for BM-MNC therapy. Moreover, ADRCs have been successfully used in several clinical situations[8-11]. This information prompts us to use ADRCs as an alternative cell source instead of BM-MNCs for the treatment of patients with CLI. To assess the safety and feasibility of intramuscular autologous ADRC implantation in patients with CLI, we initiated a clinical pilot study

Study goals and objectives

To assess the safety and feasibility of intramuscular autologous ADRC implantation in patients with CLI, we initiate a clinical pilot study. The primary outcome is the amputation-free survival ratio (AFS) during the 6-month follow-up period after the TACT-ADRC procedure. The secondary endpoints are evaluated by ABI (ankle biracial index) and skin perfusion pressure (SPP); the changes in rest pain are evaluated using the numerical rating scale (NRS). Moreover, ulcer size (grand total of the minor axis times longer axis) and walking distance in 6 min in patients with lower limb lesions are assessed. Pre-procedural and follow-up examinations at 1 and 6 months after TACT-ADRC procedure are performed. The safety of the therapy is

assessed in the context of major adverse cardiovascular events (MACEs) and all-cause adverse events during the 6-month follow-up period after the TACT-ADRC procedure. The MACEs are defined as death, non-fatal myocardial infarctions, decompensated heart failure, and stroke.

Study design

The TACT-ADRC study is an investigator-initiated, single centre, single arm, non-randomized, open-labelled clinical study conducted in Nagoya University Hospital, Japan. The target disease is CLI, which was defined as Fontaine stage III and IV due to PAD, TAO, or CDV. Patients with CLI (defined as Fontaine stages III and IV), who could not undergo surgical revascularization, qualify for ADRC implantation. All patients underwent pre-checks to determine whether they fulfilled any of the exclusion criteria: age >80 years; insufficient amount of adipose tissue; life expectancy <1 year; untreated coronary artery diseases or cerebrovascular diseases; clinical or laboratory signs of chronic or acute inflammation; a past (5 years) or current history of neoplasms, diabetes with untreated retinopathy, severe liver or kidney dysfunction, including haemodialysis, severe anemia or haematopoietic disease; pregnancy or possibility of pregnancy; or the refusal or inability to provide informed consent. We obtain written informed consent from all the patients.

Inclusion Criteria

- ✓ Target disease：PAD, TAO, and Collagen disease-associated vasculitis
- ✓ Key inclusion criteria：Patients with CLI (Fontaine Ⅲ−Ⅳ), who are not candidates for surgical revascularization

Exclusion Criteria

Patients with

- ✓ Insufficient amount of adipose tissue
- ✓ No informed consent
- ✓ Expectation of life < 1 year
- ✓ Evidence of malignant disease during the past 5 years
- ✓ Untreated coronary heart disease
- ✓ Untreated diabetic retinopathy
- ✓ Life threatening infection
- ✓ Severe liver or kidney disease
- ✓ Severe anemia or pancytopenia
- ✓ Pregnancy
- ✓ Doctor's decision not to register to this regimen

Primary outcomes

Safety and feasibility of TACT-ADRCs

- Safety: Major Cardiovascular Adverse Event (MACE)
- Efficacy: Major amputation free survival ratio, walking distance in 6 minutes, rest pain, and ischemic ulcer size

Methodology

While the patients are under general anaesthesia, about 300 ml of adipose tissue is obtained by liposuction from the subcutaneous abdominal and/or femoral sections by well-trained plastic surgeons. Concentrated ADRCs (5 ml) are automatically and aseptically isolated using a commercially available closed centrifugal cell separator system (Celution system, Cytori Therapeutics, Inc., Austin, TX, USA)[8,12] with adipose digesting enzyme product (Celase, Cytori Therapeutics, Inc.) in approximately 2 h; subsequently, total cell number and viability are counted using an automated cell counter (NucleoCounter NC-100, M&S TechnoSystems, Inc., Osaka, Japan). Isolated ADRCs are diluted with lactated Ringer's solution to a total volume of 50 ml; the diluted ADRC solution is injected into the gastrocnemius or hand of the ischaemic limb, 0.5–1.0 ml in a 3 × 3 cm grid using 26-gauge needle according to the method used for BM-MNCs implantation (50–70 sites, 1–1.5 cm deep)[13]. Antiplatelet agent intake is stopped 3–5 days before liposuction depending on the type of agent and restarted 3–5 days after cell implantation depending on the situation of haemostasis.

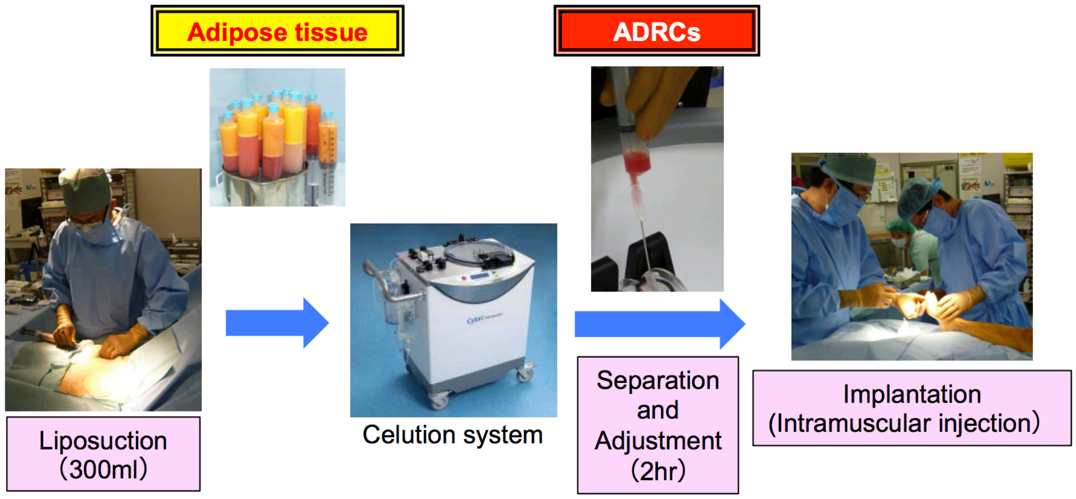

Follow-up

We have a 6-month observation period, after which we will continue regular consultations and follow up on the occurrence of adverse events.

Enrollment	Screening	TACT-ADRC	6 month follow up period
• 25 ≤ Age <80<br>• Patients with CLI	Inclusion & exclusion criteria	• Liposuction (about 300 ml)<br>• Isolation of ADRCs by celution system (Cytori Therapeutics Inc.)<br>• Transplantation of ADRC into ischemic limbs (intramuscularly)	• Safety: Major Cardiovascular Adverse Event (MACE) and all-cause adverse events.<br>• Efficacy: Amputation free survival ratio, ABI, SPP, Rest pain, Ischemic ulcer size, Walking distance in 6 minutes.

Data management and statistical analysis

One physician and one statistician with full access to the data conducted the statistical analyses. All data are expressed as the mean ± SEM. Statistical significance was evaluated using unpaired Student's t-test for comparisons between two means and repeated measures analysis of variance (ANOVA) for comparisons among three or more means, using Prism 5 (GraphPad Software). In case of a significant observation in ANOVA, Dunnett's test was performed as a post-hoc analysis. A value of $p < 0.05$ was considered statistically significant.

Quality assurance

The Celution™ system used in this study was developed by Cytori Therapeutics Inc. This system is an automated device for the isolation and concentration of cells from adipose tissue. The whole system can be operated aseptically with the use of clinical-grade solutions, such as saline and lactated Ringer's, and single-use Celution™ consumable sets.

Expected outcomes of the study

We develop a therapeutic angiogenesis procedure involving the implantation of freshly isolated autologous ADRCs in patients with CLI. Thus, we expect that autologous ADRC implantation is safe and effective for achievement of clinical therapeutic angiogenesis in patients with CLI. TACT-ADRC will improve AFS, symptoms or walking ability of patients with CLI.

Duration of the project

Patients are enrolled in this pilot study from April 2013 to November 2015.

Project management

1) Isolation of ADRCs, Transplantation of ADRC into ischemic limbs and Analyzed the data.

Takeshi Katagiri, Kazuhisa Kondo, Rei Shibata, Ryo Hayashida, Satoshi Shintani, Shukuro Yamaguchi, Yuuki Shimizu, Kazumasa Unno, Toyoaki Murohara, Ryosuke Kikuchi

2) Liposuction

Keisuke Takanari, Yuzuru Kamei

3) Surgical procedure (minor amputation etc,,)

Akio Kodama, Kimihiro Komori

Ethics

All procedures in studies involving human participants are performed in accordance with the ethical standards of the institutional or national research committee and with the 1964 Declaration of Helsinki and its later amendments or comparable ethical standards. The study protocol was approved by the Ministry of Health, Labor and Welfare in Japan and the ethics committee in Nagoya University Graduate School of Medicine, Japan in 2012. This study was registered on UMIN Clinical Trials Registry (UMIN ID: UMIN000010143; 01/03/2013). We obtain written informed consent from all the patients and their families after giving sufficient explanations for the study before the preoperative examination and providing a period of consideration.

References

1 Zuk, P. A. *et al.* Multilineage cells from human adipose tissue: implications for cell-based therapies. *Tissue Eng* **7**, 211-228, doi:10.1089/107632701300062859 (2001).

2 Zuk, P. A. *et al.* Human adipose tissue is a source of multipotent stem cells. *Mol Biol Cell* **13**, 4279-4295, doi:10.1091/mbc.E02-02-0105 (2002).

3 Nakagami, H. *et al.* Novel autologous cell therapy in ischemic limb disease through growth factor secretion by cultured adipose tissue-derived stromal cells. *Arterioscler Thromb Vasc Biol* **25**, 2542-2547, doi:10.1161/01.ATV.0000190701.92007.6d (2005).

4 Rehman, J. *et al.* Secretion of angiogenic and antiapoptotic factors by human adipose stromal cells. *Circulation* **109**, 1292-1298, doi:10.1161/01.CIR.0000121425.42966.F1 (2004).

5 Kondo, K. *et al.* Implantation of adipose-derived regenerative cells enhances ischemia-induced angiogenesis. *Arterioscler Thromb Vasc Biol* **29**, 61-66, doi:10.1161/ATVBAHA.108.166496 (2009).

6 Hao, C. *et al.* Therapeutic angiogenesis by autologous adipose-derived regenerative cells: comparison with bone marrow mononuclear cells. *Am J Physiol Heart Circ Physiol* **307**, H869-879, doi:10.1152/ajpheart.00310.2014 (2014).

7 Ishii, M. *et al.* Multilayered adipose-derived regenerative cell sheets created by a novel magnetite tissue engineering method for myocardial infarction. *Int J Cardiol* **175**, 545-553, doi:10.1016/j.ijcard.2014.06.034 (2014).

8 Gotoh, M. *et al.* Regenerative treatment of male stress urinary incontinence by periurethral injection of autologous adipose-derived regenerative cells: 1-year outcomes in 11 patients. *Int J Urol* **21**, 294-300, doi:10.1111/iju.12266 (2014).

9 Mesimaki, K. *et al.* Novel maxillary reconstruction with ectopic bone formation by GMP adipose stem cells. *Int J Oral Maxillofac Surg* **38**, 201-209, doi:10.1016/j.ijom.2009.01.001 (2009).

10 Yoshimura, K. *et al.* Progenitor-enriched adipose tissue transplantation as rescue for breast implant complications. *Breast J* **16**, 169-175, doi:10.1111/j.1524-4741.2009.00873.x (2010).

11 Panes, J. *et al.* Expanded allogeneic adipose-derived mesenchymal stem cells (Cx601) for complex perianal fistulas in Crohn's disease: a phase 3 randomised, double-blind controlled trial. *Lancet* **388**, 1281-1290, doi:10.1016/S0140-6736(16)31203-X (2016).

12 Lin, K. *et al.* Characterization of adipose tissue-derived cells isolated with the Celution system. *Cytotherapy* **10**, 417-426, doi:10.1080/14653240801982979 (2008).

13 Tateishi-Yuyama, E. *et al.* Therapeutic angiogenesis for patients with limb ischaemia by autologous transplantation of bone-marrow cells: a pilot study and a randomised controlled trial. *Lancet* **360**, 427-435, doi:10.1016/S0140-6736(02)09670-8 (2002).
