# Supplementary material for: Therapeutic angiogenesis using autologous adipose-derived regenerative cells in patients with critical limb ischaemia in Japan: a clinical pilot study
Source: Sci Rep. 2020 Sep 29;10:16045. doi: 10.1038/s41598-020-73096-y (PMC7525513; doi:10.1038/s41598-020-73096-y)

Supplementary Information file

Therapeutic Angiogenesis using Autologous Adipose-derived Regenerative Cells in Patients with Critical Limb Ischaemia in Japan: A Clinical Pilot Study

Takeshi Katagiri[1], Kazuhisa Kondo[1], Rei Shibata[2], Ryo Hayashida[1], Satoshi Shintani[1], Shukuro Yamaguchi[1], Yuuki Shimizu[1], Kazumasa Unno[1], Ryosuke Kikuchi[3], Akio Kodama[4], Keisuke Takanari[5], Yuzuru Kamei[5], Kimihiro Komori[4] and Toyoaki Murohara[1]

[1] Department of Cardiology, Nagoya University Graduate School of Medicine, Nagoya, Japan

[2] Department of Advanced Cardiovascular Therapeutics, Nagoya University Graduate School of Medicine, Nagoya, Japan

[3] Department of Medical Technique, Nagoya University Hospital, Japan.

[4]Department of Vascular Surgery, Nagoya University Graduate School of Medicine, Nagoya, Japan

[5] Department of Plastic and Reconstructive Surgery, Nagoya University Graduate School of Medicine, Nagoya, Japan

Figure 5a

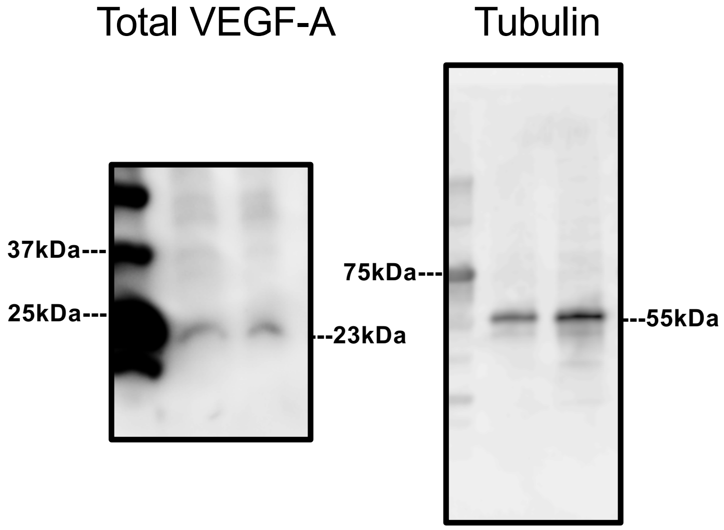

Figure 5b

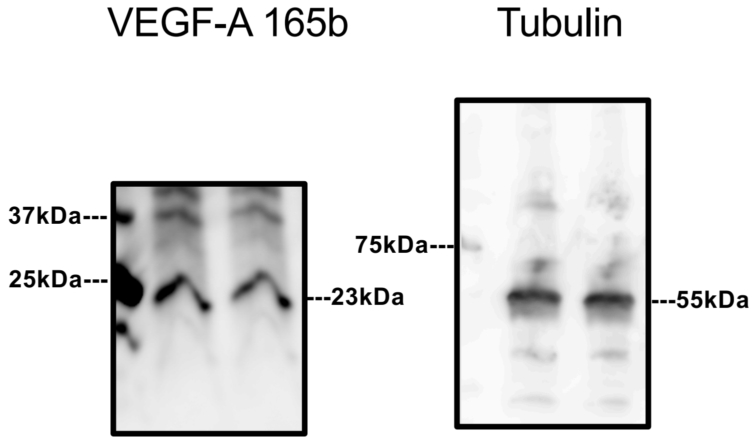

Figure 5e

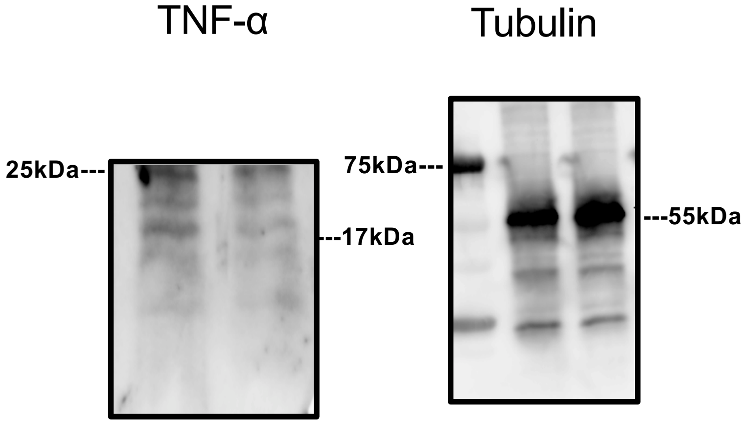

Figure 6a

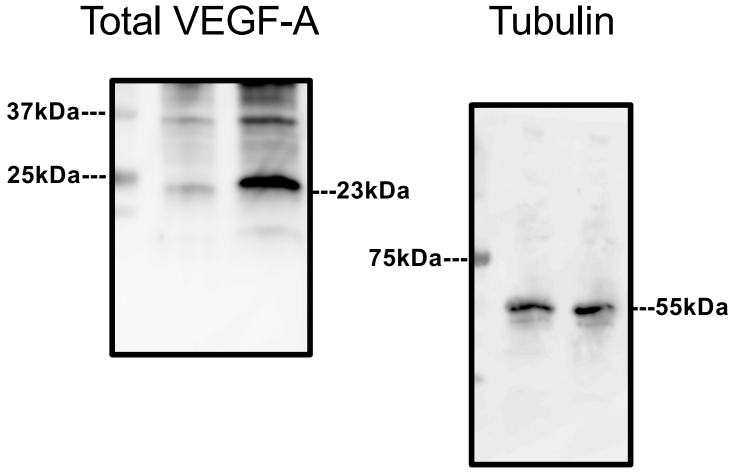

Figure 6b

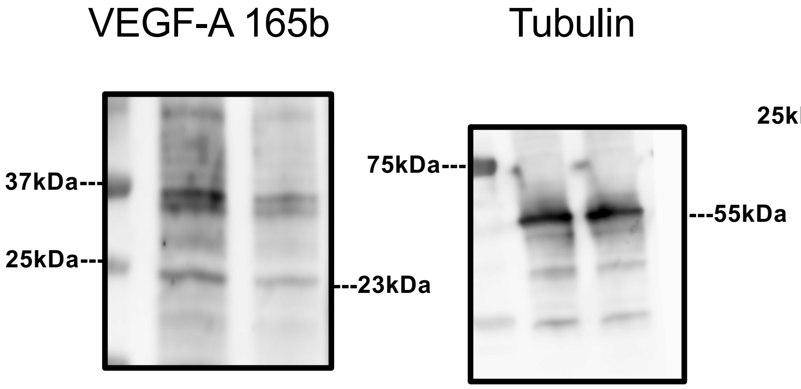

Figure 6c

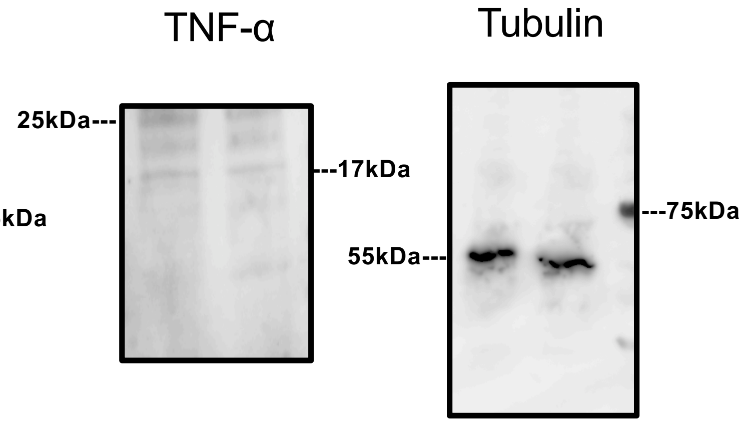

Supplement: Supplementary file 2 [file 41598_2020_73096_MOESM2_ESM.pdf]
